# Busulfan plus fludarabine as a myeloablative conditioning regimen compared with busulfan plus cyclophosphamide for acute myeloid leukemia in first complete remission undergoing allogeneic hematopoietic stem cell transplantation: a prospective and multicenter study

**DOI:** 10.1186/1756-8722-6-15

**Published:** 2013-02-08

**Authors:** Hui Liu, Xiao Zhai, Zhaoyang Song, Jing Sun, Yang Xiao, Danian Nie, Yu Zhang, Fen Huang, Hongsheng Zhou, Zhiping Fan, Sanfang Tu, Yonghua Li, Xutao Guo, Guopan Yu, Qifa Liu

**Affiliations:** 1 Department of Hematology, Nanfang Hospital, Southern Medical University, Guangzhou, 510515, China; 2 Department of Hematology, Zhujiang Hospital, Southern Medical University, Guangzhou, 510282, China; 3 Department of Hematology, Guangzhou General Hospital of Guangzhou Military Command, Guangzhou, China; 4 Department of Hematology, Sun Yat-Sen Memorial Hospital, Sun Yat-Sen University, Guangzhou, Guangdong, China

**Keywords:** Busulfan, Fludarabine, Allogeneic hematopoietic stem cell transplantation

## Abstract

**Objective:**

We conducted a prospective, randomized, open-label, multicenter study to compare busulfan plus fludarabine (BuFlu) with busulfan plus cyclophosphamide (BuCy) as the conditioning regimen in allogeneic hematopoietic stem cell transplantation (allo-HSCT) for acute myeloid leukemia (AML) in first complete remission (CR1).

**Methods:**

Totally 108 AML-CR1 patients undergoing allo-HSCT were randomized into BuCy (busulfan 1.6 mg/kg, q12 hours, -7 ~ -4d; cyclophosphamide 60 mg/kg.d, -3 ~ -2d) or BuFlu (busulfan 1.6 mg/kg, q12 hours, -5 ~ -2d; fludarabine 30 mg/m^2^.d, -6 ~ -2d) group. Hematopoietic engraftment, regimen-related toxicity (RRT), graft-versus-host disease (GVHD), transplant related mortality (TRM), and overall survival were compared between the two groups.

**Results:**

All patients achieved hematopoietic reconstitution except for two patients who died of RRT during conditioning. All patients obtained complete donor chimerism by day +30 post-transplantation. The incidence of total and III-IV RRT were 94.4% and 81.5% (*P* = 0.038), and 16.7% and 0.0% (*P* = 0.002), respectively, in BuCy and BuFlu group. With a median follow up of 609 (range, 3–2130) days after transplantation, the 5-year cumulative incidence of TRM were 18.8 ± 6.9% and 9.9 ± 6.3% (*P* = 0.104); the 5-year cumulative incidence of leukemia relapse were 16.5 ± 5.8% and 16.2 ± 5.3% (*P* = 0.943); the 5-year disease-free survival and overall survival were 67.4 ± 7.6% and 75.3 ± 7.2% (*P* = 0.315), and 72.3 ± 7.5% and 81.9 ± 7.0% (*P* = 0.177), respectively in BuCy and BuFlu group.

**Conclusion:**

Compared with BuCy, BuFlu as a myeloablative condition regimen was associated with lower toxicities and comparable anti-leukemic activity in AML-CR1 patients undergoing allo-HSCT.

## Background

Acute myeloid leukemia (AML) is one of the most common leukemias with a 20% 5-year event-free survival in adults. The incidence of AML increases sharply with age [1]. Allogeneic hematopoietic stem-cell transplantation (allo-HSCT) is an effective and potentially curative treatment for AML [2,3]. The conditioning regimen with busulfan plus cyclophosphamide (BuCy) is considered as the standard myeloablative regimen for AML. However, it is associated with significant risks of regimen-related toxicity (RRT) as well as transplant related mortality (TRM), especially for the elderly and those with comorbidities. Although the introduction of intravenous busulfan has been associated with a decreased incidence of RRT, the incidence of fatal hepatic veno-occlusive disease (HVOD) remains high. Interactions between busulfan and cyclophosphamide might result in increased liver toxicity [4]. Recently, to decrease the incidence of RRT and TRM, fludarabine, a strongly immunosuppressive purine analog with considerable anti-neoplastic and immunosuppressive activity, was selected to replace cyclophosphamide in myeloablative and non-myeloablative conditioning regimens [5-12]. Non-randomized comparisons showed that myeloablative regimen based on busulfan and fludarabine (BuFlu) might be associated with limited extra-hematologic toxicities, sufficient anti-leukemic effects, better overall survival (OS) and disease free survival (DFS) for patients undergoing HSCT compared with BuCy [8-10]. However, some studies have documented that BuFlu was associated with an increased risk of relapse compared with BuCy regimen, especially in patients with active disease at the time of transplantation [13-15]. Moreover, a recent randomized comparison trial suggested that BuFlu regimen had significantly lower OS and DFS than BuCy regimen for allo-HSCT in patients with leukemia and myelodysplastic syndrome (MDS). This randomized comparison trial enrolled a heterogeneous group of patients including patients with myeloid and lymphoid neoplasms, cases in complete remission (CR) or non-remission (NR) before transplantation [14]. There have been no randomized studies comparing the toxicity and effectiveness of BuCy and BuFlu as myeloablative conditioning regimens in patients with a single disease and having a similar, balanced, disease status at the time of transplantation.

In this study, we performed a prospective, randomized, open-labeled, multicenter trial to compare the safety and efficacy of BuCy and BuFlu myeloablative conditioning regimens in patients undergoing allo-HSCT for AML in first CR (CR1).

## Materials and methods

### Study design

This prospective, randomized, open-label, multicenter study was conducted from January 2007 to May 2012 in Nanfang Hospital, Southern Medical University; Zhujiang Hospital, Southern Medical University; Guangzhou General Hospital of Guangzhou Military Command, and Sun Yat-Sen Memorial Hospital, Sun Yat-Sen University. The study was performed in accordance with the modified Helsinki Declaration. The protocol was approved by respective ethical review boards.

### Patients

Eligibility criteria included patients with AML-CR1 and age between 12 and 60 years. Exclusion criteria included the following: ① acute promyelocytic leukemia; ② patients with secondary leukemia; ③ more than 3 cycles of induction chemotherapy; ④ systolic ejection fraction less than 30%; ⑤ abnormal pulmonary spirometry test (percentage of the predicted value of forced expiratory volume in first second less than 60% and/or percentage of the predicted value of pulmonary diffusion capacity less than 60%); ⑥ serum bilirubin or alanine aminotransferase and/or aspartate aminotransferase two times more than the upper limit of normal; ⑦ creatinine clearance less than 50 ml/min. Written informed consent was obtained from each recipient and donor.

### Conditioning regimen

Eligible patients were randomized to receive BuCy or BuFlu conditioning regimen using a permuted block randomization. In the BuCy group, busulfan was administered continuously for 4 hours through a central venous catheter at 1.6 mg/kg every 12 hours on days −7 to −4, and cyclophosphamide 60 mg/kg.d was given daily on days −3 to −2. In the BuFlu group, busulfan was given at the same dose and schedule, and fludarabine 30mg/m^2^.d was given continuously for 1 hour once daily after the first dose of busulfan on days −6 to −2.

### HLA typing and source of donor

Genomic high-resolution molecular typing was used for HLA-A, -B, -C, -DRB1 and -DRQ. Ninety-four cases were HLA-matched and 14 were HLA-mismatched. Ninety three patients were transplanted from related donors (siblings in 89 cases and family members in 4 cases), and 15 patients from unrelated donor. Ninety eight patients received peripheral blood stem cells (PBSCs) grafts, and 10 received bone marrow cells and PBSCs mixed grafts.

### Graft-versus-host disease (GVHD) and infections prophylaxis

Cyclosporine A (CsA, 1-3 mg/kg.d, with serum valley value maintained at 200-300 ng/ml) plus methotrexate (MTX, 15 mg on days +1 and 10 mg on days +3 and +6) were administered in the patients undergoing HLA matched sibling donor transplants for GVHD prophylaxis. CsA, MTX and mycophenolate mofetil (MMF, 0.5 g twice a day on days 0 to +28) were used in the patients undergoing 1 locus HLA-mismatched sibling donor transplantation, and CsA, MTX and human anti-thymocyte globulin (ATG [Thymoglobulin; Genzyme, Cambridge, MA]; 2.5 mg/kg.d on days −3 to −1) in the patients undergoing HLA-matched unrelated donor, or more than 1 locus HLA-mismatched sibling donor transplantation. The patients undergoing HLA-mismatched unrelated donor or haploidentical transplantation received CsA + MTX and ATG + MMF as GVHD prophylaxis [16].

Oral sulfamethoxazole and norfloxacin were given to all patients. Acyclovir was given daily from the beginning of preparation therapy to engraftment, and for 7 days every 2 weeks up to 1 year after transplantation. Ganciclovir was used for 2 weeks before transplantation for prophylaxis of cytomegalovirus (CMV) infection, and used again during CMV viremia periods within 1 year post-transplantation. Antifungal agents were administered 5 days pre-transplantation for +30 to +60 days in patients with no history of invasive fungal infection (IFI), and at least 90 days post-transplantation in those with a history of IFI [16].

### Monitoring of epstein barr virus (EBV) -DNA and CMV-DNA levels in blood

Generally, the EBV-DNA and CMV-DNA levels in blood were monitored weekly for three months after transplantation. During the 4th to 9th month post-transplantation, the monitoring frequency was once every two weeks; the 10th to the 24th month, once a month; the 25th to 36th month, once every three months. If EBV-DNA or CMV-DNA was positive, it was monitored twice a week. The DNA levels of EBV and CMV in blood were detected by quantitative real-time polymerase chain reaction (RQ-PCR) according to our method previously described [17].

### Supportive care

Low molecular weight heparin (Fraxiparine, 0.6 mg i.v. continuously for 24 h) and prostaglandin E (Alprostadil, 10 mg, 3 times per day) were used from the beginning of the conditioning to engraftment for HVOD prophylaxis. Phenytoin (400 mg, 3 times per day) orally was used from 1 day before intravenous busulfan therapy to 48 h after the discontinuation of busulfan for the prophylaxis of busulfan toxicities on central nervous system. All patients received granulocyte colony-stimulating factor (G-CSF, 5 μg/kg.d) from +3 days post-transplantation until achievement of the peripheral white blood cells count reached 1.0 × 10^9^/L or absolute neutrophil count (ANC) reached 0.5 × 10^6^/L. Patients received red blood cells and platelet transfusions if hemoglobin levels were ≤70 g/L and platelet count ≤20.0 × 10^9^/L.

### Analysis of chimerism

Bone marrow donor-recipient chimerism on days +15 and +30 after transplantation was evaluated using fluorescein in situ hybridization (FISH) in sex-mismatched transplantation and short tandem repeat analysis in sex-matched transplantation.

### Endpoints and definitions

The primary endpoint was RRT, and secondary endpoints were hematopoietic engraftment, bone marrow toxicities, the incidence and severity of acute and chronic GVHD, early infections, disease relapse, TRM, OS and DFS after transplantation. RRT was graded according to Bearman’s criteria [18]. Hematopoietic engraftment was defined as the first of 2 consecutive days with an ANC in the peripheral blood exceeding 0.5 × 10^9^/L and the first of 3 days with an absolute platelet count exceeding 20 × 10^9^/L without transfusion support. Complete chimerism was defined as >95% donor cells detected; mixed chimerism, as 5% to 95% donor cells detected [16]. Acute GVHD (aGVHD) was graded according to standard criteria, and chronic GVHD (cGVHD) was assessed in patients alive after day 100 and defined as limited or extensive [19,20]. Hematologic relapse was defined by reappearance of blasts in the blood, any manifestation of leukemia outside the hematopoietic system, or greater than 5% blasts in the bone marrow smear. Genetic relapse was assessed by chimerism status and presence of the tumor target gene marker, and relapse was defined as the rate of donor chimerism status decreasing more than 5% or the reappearance of the tumor target gene marker. TRM was defined as death from any cause other than relapse. DFS was defined as survival in a state of continuous complete remission.

### Statistics

All patients were studied in their assigned treatment group on an intent-to-treat analysis. Analysis was performed on October 31, 2012. Comparisons of categorical variables were made by means of chi-squared and Fisher exact tests for small numbers. Differences between numerical variables were calculated by means of 2-sample t test. Incidence of time-dependent variables was estimated by the method of Kaplan-Meier. The Cox’ regression model was used for analyzing prognostic factors for relapse, DFS, and OS. Numerical variables were analyzed as categories based on their values being below or above the median of the entire cohort. Patient’s age, gender, pre-transplantation chemotherapy cycles, genetic subgroups, HLA-typing, donor age, the source of grafts, conditioning regimen, mononuclear cell dose, CD34^+^ cell dose, aGVHD, and cGVHD were analyzed in univariate and multivariate analysis. Intervals were measured from the day of transplantation until the last day of follow-up, transplant-related death or relapse. The SPSS software package (SPSS, Chicago, IL) was used for all data analysis. All statistical tests were two-sided, and P-value less than 0.05 was used to indicate statistical significance. The study was originally designed for 110 patients in order to detect a 10% decrease in RRT with an α error of 0.05 and a β error of 0.10.

## Results

### Patient and transplant characteristics

At the time when the study was closed, a total of 110 patients were enrolled and two patients were eliminated before randomization because of disease relapse before transplantation. A hundred and eight patients were randomized to BuCy group (n = 54) or BuFlu group (n = 54). The median age was 30.5 years (range 12–54 years), with 56 males and 52 females. Genetic data was available in 90 patients. The genetic subgroups according to the criteria [21] were favorable in 11 cases, intermediate in 49 cases, and unfavorable in 30 cases . Within the study population, 4 patients had comorbidities: 1 had hypertension, 1 had diabetes mellitus, 1 had diabetes mellitus and hypertension, and 1 had Gilbert’s syndrome and chronic bronchitis. Two patients had a history of pulmonary tuberculosis and 28 had a history of IFI (including stable in 21 cases, and active in 7 cases) at the time of transplantation. Characteristics of patients, donors and transplants are summarized in Table 1. There were no significant differences in patients’ age, gender, induction and consolidation chemotherapy cycles, genetic subgroups, HLA-typing, and the source of donors and grafts between the two groups (Table 1, all *P* > 0.05). 

**Table 1:** The characteristics of the patients

	***BuCy***	***BuFlu***	***P value***
No. of patients	54	54	
Sex male/female	27/27	25/29	0.700
Median age in years (range)	30.5(12 ~ 52)	30.5(14 ~ 54)	0.518
Genetic subgroups favorable/intermediate/poor risk/unknown	2/24/16/12	9/25/14/6	0.085
Induction chemotherapy cycles (range)	1 (1–3)	1 (1–3)	0.056
Consolidation chemotherapy cycles (range)	2.5 (1–4)	2 (1–4)	0.222
Source of donors related/unrelated	47/7	45/9	0.588
HLA typing matched/mismached	48/6	46/8	0.567
Source of stem cells PB /BM + PB	48/6	50/4	0.507
Cell yield (median) MNC(10^8^/Kg)	7.87	7.22	0.510
CD34 + (10^6^/Kg)	7.30	5.92	0.112

Abbreviations: *BuCy*, busulfan-cyclophosphamide; *BuFlu*, busulfan-fludarabine;

*BM*, bone marrow; *PB*, peripheral blood; *MNC*, mononucleated cell.

### Engraftment and chimerism

All patients achieved hematopoietic reconstitution except two patients who died of RRT during conditioning. The median time to neutrophil engraftment were 12 (range, 9–19 days) and 11 days (range, 9–15 days), respectively, in BuCy and BuFlu group (*P* = 0.056). The median time to platelet engraftment were 13 (range, 9–35 days) and 12 days (range, 9–45 days), respectively, in BuCy and BuFlu group (*P* = 0.562). All 106 evaluable patients achieved donor chimerism, including 24 cases with complete chimerism and 30 cases with mixed donor chimerism (with donor chimerism ranging from 78% to 93%) in BuCy group, and 23 cases with complete chimerism and 31 cases with mixed donor chimerism (with donor chimerism ranging from 68% to 92%) in BuFlu group (*P* = 0.845) by day 15 after transplantation. All patients achieved complete donor chimerism by day 30 after transplantation.

### Bone marrow toxicities

The median duration of neutrophil count below 0.1 × 10^9^/L were 4 (range, 1–14 days) and 3 days (range, 0–9 days), respectively, in BuCy and BuFlu group (*P* = 0.008). The median duration of platelet count below 20 × 10^9^/L were 5 (range, 1–21 days) and 3 days (range, 0–43 days), respectively, in BuCy and BuFlu group (*P* = 0.043). The median number of the red blood cell transfusion were 2 (range, 0–7u) and 1u (range, 0–8u), respectively, in BuCy and BuFlu group (*P* = 0.007). The median number of the platelet concentrates transfusion were 3 (range, 0–19u) and 2u (range, 0–11u), respectively, in BuCy and BuFlu group (*P* = 0.028).

### RRT

The most common toxicity was mucositis (60/108). The incidence of total RRT were 94.4% and 81.5% (*P* = 0.038), and III-IV RRT were 16.7% and 0.0% (*P* = 0.002), respectively, in BuCy and BuFlu group. There were four regimen-related deaths in BuCy group, including 1 who died from delayed hemorrhagic cystitis, 1 who died from HVOD and 2 who died from acute heart failure; no patient died from RRT in BuFlu group (*P* = 0.042). In addition, autopsy of one patient that died from cardiac toxicity showed limited myocardial fibrosis. Organ toxicities are summarized in Table 2.

**Table 2:** Organ toxicity according to Bearman’s criteria

	**Grade I or II**		**Grade III or IV**		
	**BuCy**	**BuFlu**	**BuCy**	**BuFlu**	**P value**
Heart (single/multiple)	4/8	2/4	2/0	0/0	0.048
Bladder	5/8	2/4	0/1	0/0	0.010
Kidneys	0/2	0/1	0/0	0/0	0.500
Lungs	0/0	0/0	0/0	0/0	
Liver	4/10	4/5	0/1	0/0	0.165
CNS	0/0	0/1	0/3	0/0	0.618
Mucosa	16/20	15/9	0/0	0/0	0.020
Gut	1/24	10/6	0/2	0/0	0.018

Abbreviations: *BuCy*, busulfan-cyclophosphamide; *BuFlu*, busulfan-fludarabine; *CNS*, central nervous system.

### Infections post-transplantation

Within the first 100 days after transplantation, 67 patients developed 90 episodes of bacterial and/or fungal infections. There were 14 and 12 cases of bacterial infections, 8 and 7 cases of fungal infections, and 14 and 12 cases of bacterial and fungal mixed infections, respectively, in BuCy and BuFlu group (*P* = 0.805). One patient died from bacterial infection in BuFlu group, while no patient died from bacterial or fungal infections in BuCy group (*P* = 1.000). In addition, within the 6 months of transplantation, 11 patients in BuCy group and 10 patients in BuFlu group had CMV viremia (*P* = 0.808); 4 patients in BuCy group and 4 patients in BuFlu group had EBV viremia (*P* = 1.000). With a median follow up of 609 days after transplantation, CMV associated diseases occurred in 2 patients in BuCy group and 1 patient in BuFlu group (CMV pneumonia, *P* = 1.000); EBV associated diseases occurred in 4 patients in BuCy group [post-transplant lymphoproliferative disease (PTLD) in 3, and EBV associated fever in 1] and 3 patients in BuFlu group (PTLD in 1, EBV associated fever in 1, and encephalitis in 1) (*P* = 1.000). One patient died from CMV pneumonia in BuCy group and 2 patients died from EBV and CMV associated diseases in BuFlu group (EBV encephalitis in 1 and CMV pneumonia in 1).

### GVHD

The incidence of I-II° and III-IV° aGVHD were 53.8% and 7.7%, and 44.4% and 0.0%, respectively, in BuCy and BuFlu group (*P* = 0.333 and *P* = 0.054, respectively). cGVHD occurred in 24 of 51 (47.1%) and 24 of 54 (44.4%) patients, respectively, in BuCy and BuFlu group (*P* = 0.788). And the incidence of extensive cGVHD were 19.6% and 14.8%, respectively, in BuCy and BuFlu group (*P* = 0.515). Two patients died from cGVHD in BuCy group, while no patients died from GVHD in BuFlu group.

### Relapse

**Figure 1:**
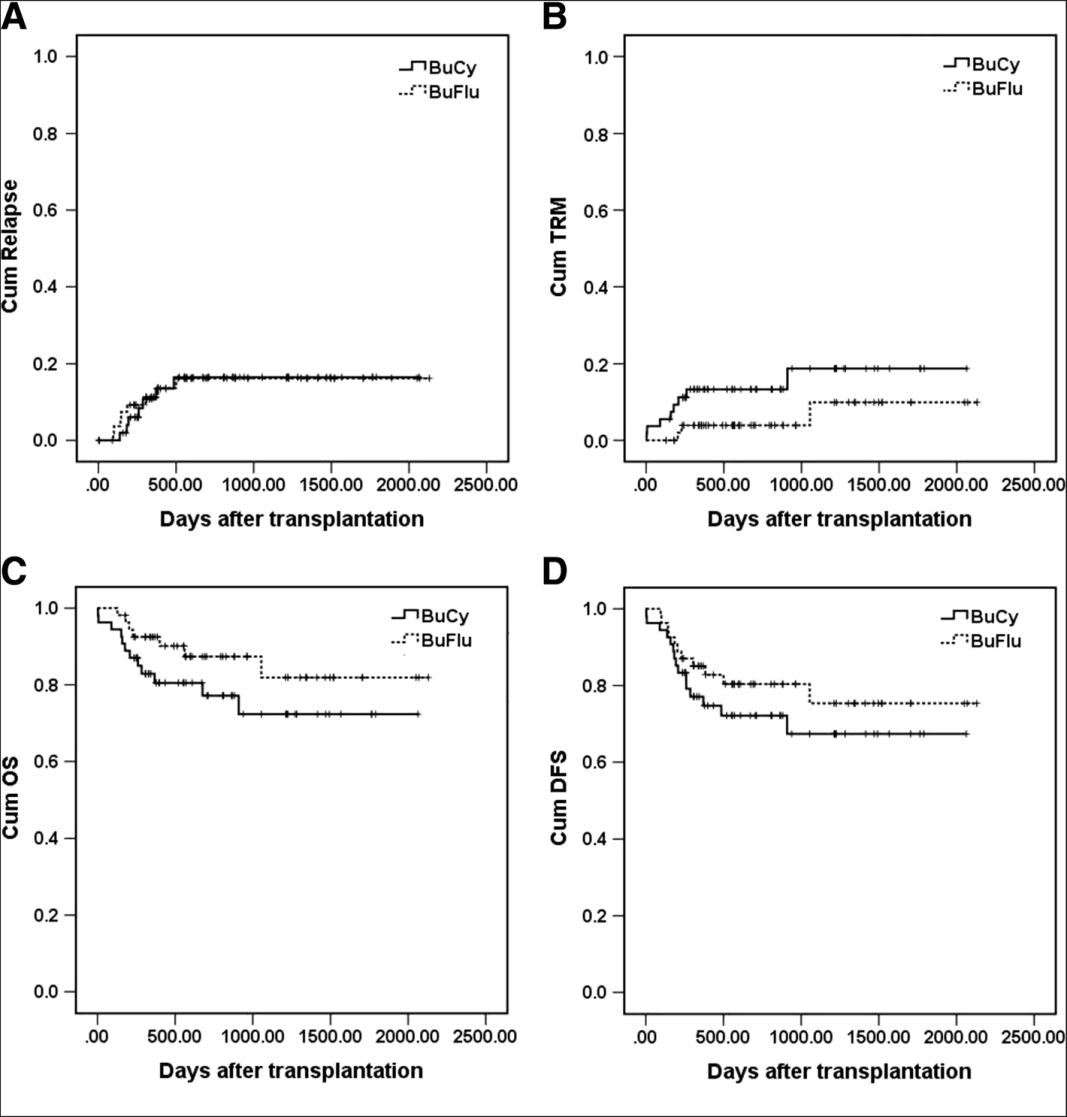
**Cumulative incidence of relapse (A), transplant related mortality (TRM) (B), overall survival (OS) (C) and disease free survival (DFS) (D).** The 5-year cumulative incidence of relapse were 16.5 ± 5.8% and 16.2 ± 5.3% in BuCy and BuFlu group (*P* = 0.943). The 5-year cumulative incidence of TRM were 18.8 ± 6.9% and 9.9 ± 6.3% in BuCy and BuFlu group (*P* = 0.104). The 5-year cumulative OS were 72.3 ± 7.5% and 81.9 ± 7.0%, respectively, in BuCy and BuFlu group (*P* = 0.177), and DFS were 67.4 ± 7.6% and 75.3 ± 7.2%, respectively, in BuCy and BuFlu group (*P* =0.315)

During our study, 15 patients relapsed (13.0%): hematologic relapse was detected in 6 and 6 patients, and genetic relapse occurred in 1 and 2 patients, respectively, in BuCy and BuFlu group (*P* = 0.781). The 5-year cumulative incidence of relapse were 16.5 ± 5.8% and 16.2 ± 5.3% in BuCy and BuFlu group (Figure 1A, *P* = 0.943). Of the 15 relapse patients, 3 patients abandoned treatment and the other 12 patients were treated with chemotherapy, donor lymphocyte infusion or second allo-HSCT. Seven cases achieved CR after treatment. In the univariate analysis, genetic subgroups and HLA-typing were significantly associated with disease relapse (*P* = 0.002 and *P* = 0.011, respectively). In the multivariate analysis, higher genetic risk (relative risk [RR] 2.218, CI 1.146–4.293, *P* = 0.018) was significantly associated with a higher relapse rate (Table 3).

**Table 3:** Risk factors for OS, DFS, TRM and relapse incidence

	**OS**		**DFS**		**TRM**		**Relapse rate**	
	**Univariate**	**Multivariate (RR)**	**Univariate**	**Multivariate (RR)**	**Univariate**	**Multivariate (RR)**	**Univariate**	**Multivariate (RR)**
Sex male/female	NS	NS	NS	NS	NS	NS	NS	NS
Genetic subgroups favorable/intermediate/poor risk/unknown	NS	NS	NS	NS	NS	NS	*P* = 0.002	*P* = 0.018 (2.218)
Age ≤30.5/>30.5 years	NS	NS	NS	NS	NS	NS	NS	NS
Induction Chemotherapy cycles ≤1/>1 cycles	NS	NS	NS	NS	NS	NS	NS	NS
Consolidation chemotherapy cycles ≤2/>2 cycles	NS	NS	NS	NS	NS	NS	NS	NS
Source of stem cells PB /BM + PB	NS	NS	NS	NS	NS	NS	NS	NS
Source of donors related/unrelated	NS	NS	NS	NS	NS	NS	NS	NS
Age of donors ≤30.0/>30.0 years	NS	NS	NS	NS	*P* = 0.026	NS	NS	NS
HLA typing matched/mismached	NS	NS	NS	NS	NS	NS	*P* = 0.011	NS
Conditioning regimen BuCy/BuFlu	NS	NS	NS	NS	NS	NS	NS	NS
MNC(10^8^/Kg) ≤7.33/>7.33	NS	NS	NS	NS	NS	NS	NS	NS
CD34 + (10^6^/Kg) ≤6.22/>6.22	NS	NS	NS	NS	NS	NS	NS	NS
Acute GVHD With/without	*P* = 0.014	*P* = 0.018 (5.214)	NS	NS	*P* = 0.001	*P* = 0.006 (18.538)	NS	NS
Chronic GVHD With/without	NS	NS	NS	NS	NS	NS	NS	NS

Abbreviations: *OS*, overall survival; *DFS*, disease free survival; *TRM*, transplant related mortality; *BuCy*, busulfan-cyclophosphamide; *BuFlu*, busulfan-fludarabine; *BM*, bone marrow; *PB*, peripheral blood; *MNC*, mononucleated cell.

### OS and DFS

With a median follow up time of 609 days (range, 3–2130) after transplantation, 89 patients were alive and 19 patients died. TRM occurred in 8 patients (RRT in 4, cGVHD in 2, thrombotic microangiopathy in 1, and CMV pneumonia in 1) in BuCy group, and 3 patients (bacterial infection in 1, EBV encephalitis in 1 and CMV pneumonia in 1) in BuFlu group (*P* = 0.112). The 5-year cumulative incidence of TRM were 18.8 ± 6.9% and 9.9 ± 6.3% in BuCy and BuFlu group (Figure 1B, *P* = 0.104). The 5-year cumulative OS were 72.3 ± 7.5% and 81.9 ± 7.0%, respectively, in BuCy and BuFlu group (Figure 1C, *P* = 0.177), and DFS were 67.4 ± 7.6% and 75.3 ± 7.2%, respectively, in BuCy and BuFlu group (Figure 1D, *P* = 0.315). In the univariate analysis, aGVHD was significantly associated with OS (*P* = 0.014) and TRM (*P* = 0.001), and age was significantly associated with TRM (*P* = 0.026). In the multivariate analysis, severe aGVHD was significantly associated with a lower OS (RR 5.214, CI 1.333–20.404, *P* = 0.018), and a higher TRM (RR 18.538, CI 2.280–150.723, *P* = 0.006). All factors studied were not significantly associated with DFS in the univariate and multivariate analysis (Table 3).

## Discussion

BuCy is considered as a standard conditioning regimen for allo-HSCT in AML patients. The introduction of i.v. busulfan decreases the intra- and inter-individual variability in systemic busulfan exposure, improving the safety of BuCy regimen. However, early RRT was still substantial, especially HVOD. Busulfan and cyclophosphamide are mainly metabolized in liver. Both toxic cyclophosphamide metabolites and busulfan can decrease the levels of glutathione (GSH) [4,22]. Combination of these two alkylating agents may result in an exacerbated risk for serious hepatic injuries [4]. Recently, to limit RRT and TRM, non-myeloablative and reduced-intensity conditioning regimens are increasingly used, however, relapse becomes more prevalent. Therefore, the challenge is to develop myeloablative regimens associated with low TRM and sufficient anti-leukemic effect.

Fludarabine is widely used in chemotherapy of acute leukemia (especially refractory leukemia) and in conditioning regimens for allo-HSCT. Fludarabine has a synergistic interaction with busulfan through inhibition of DNA ligase and DNA primase, and prevention of DNA polymerization, impairing alkylator-induced damage repair. In addition, fludarabine does not depend on hepatic GSH-stores for its detoxification. Thus, there are non-overlapping organ toxicities between fludarabine and busulfan. A growing body of evidence suggests that fludarabine plus busulfan as myeloablative or non-myeloablative conditioning regimen results in a favorable balance between anti-malignancy efficacy and reduced toxicities [5-12]. In this study, we observed that the incidence of RRT and regimen-related death were significantly higher in BuCy regimen compared with BuFlu regimen in patients with AML-CR1 undergoing allo-HSCT. BuCy regimen showed higher incidence of bladder, mucosa and gut adverse events compared with BuFlu regimen. These results were consistent with previous studies [8-10]. Surprisingly, we observed that 2 patients died from heart toxicities and autopsy of one case showed limited myocardial fibrosis, suggesting that BuCy might be more toxic to the heart than BuFlu, especially to those with a history of heart disease. Although total hepatic toxicities were similar between the two regimens, one patient died of HVOD in BuCy regimen in our study. No differences in TRM between the two regimens might be attributed to the young age and lack of comorbidities of patients in our cohort. We also observed that the median duration of neutrophil count below 0.1 × 10^9^/L and platelet count below 20 × 10^9^/L in BuCy regimen were significantly longer than that in BuFlu regimen. Meanwhile, BuCy regimen required more red blood cells and platelet concentrates transfusions compared with BuFlu regimen. From these data, it can be suggested that BuFlu regimen might possess less RRT and bone marrow toxicities as well as safer than BuCy regimen. In addition, Russell et al. reported that single daily intravenous busulfan would be more convenient and could be achieved with acceptable toxicity compared with traditional 4-times-daily dosing. In this report, busulfan was administered every 12 hours and associated with higher peak blood concentration after the first day and similar toxicity compared with 4-times-daily dosing (data not shown). Whether this 2-times-daily dosing is associated with increased anti-leukemic activity remains to be discussed.

Other than relapse of malignancy, GVHD and infections remain as the two main causes of death after allo-HSCT. A shorter duration of neutropenia and faster hematopoietic recovery may reduce risk of infection after transplants [23,24]. In the present study, the bone marrow suppression toxicities were lower in BuFlu regimen than BuCy regimen. However, the incidence of early infectious post-transplantation (including bacterial and fungal infections) did not differ between the two regimens, probably as a consequence of almost all patients being treated in a sterile ward for 100 days after transplantation and the short duration of neutropenia. Some studies suggested that the conditioning regimens containing fludarabine were associated with higher incidence of opportunistic infections such as CMV and EBV after transplantation because of the immunosuppressive effect of fludarabine [10,25,26]. Here, we observed that the incidence of CMV and EBV viremia was similar between BuFlu and BuCy regimens within 6 months after transplantation. During the follow-up period, there were also no differences in the incidence of CMV and EBV-associated diseases between the two regimens.

GVHD is a complex pathological process mediated by allo-reactive donor T cells recognizing the disparate HLA antigens and involving tissue-specific immune cells and inflammatory cytokines [27]. The incidence and severity of GVHD can be influenced by many factors including age of recipient and donor, HLA typing, source of donor and stem cells, and conditioning regimens. The conditioning can provoke the release of inflammatory factors, which play critical roles in aGVHD [28]. Chae et al. reported that the incidence and severity of acute and chronic GVHD were lower in BuFlu regimen compared with BuCy regimen [9]. However, in a randomized trial, Lee et al. suggested that the incidence and severity of acute and chronic GVHD were similar between the two regimens [14]. In this report, the incidence of I-II° and III-IV° aGVHD as well as total and extensive cGVHD were also similar between BuFlu and BuCy regimens.

Some single arm and retrospective comparison studies suggested that myeloablative BuFlu regimen did not increase the risk of disease relapse [9,10]. However, Shimoni et al. reported a non-statistically significant trend for higher incidence of relapse after BuFlu myeloablative regimen [13]. Lee et al. reported that BuFlu regimen had lower relapse-free survival than BuCy regimen [14]. However, these two studies enrolled patients with different diseases and disease status before transplantation. In this study, we found no significant difference in relapse rate between BuCy and BuFlu regimens for AML-CR1 patients. Based on these results, we concluded that BuFlu and BuCy had equivalent anti-leukemic activity to AML-CR1 patients undergoing allo-HSCT.

The survival of patients with AML after allo-HSCT is influenced by many factors, such as the response to induction therapy, white blood cell count at diagnosis, cytogenetics, the status of disease during transplantation, and conditioning regimens [29,30]. Several single arm and retrospective comparisons showed that myeloablative regimen based on busulfan and fludarabine might be associated with fewer RRT, lower TRM, and higher survival rates compared with BuCy [8-10]. However, a recent randomized prospective trial from Lee et al. reported that BuCy regimen had better 2-year OS and DFS than BuFlu in patients with leukemia and MDS [14]. In the present randomized prospective trial, the 5-year cumulative OS and DFS were 81.9 ± 7.0% and 75.3 ± 7.2% in AML-CR1 patients receiving BuFlu, and were not different from patients receiving BuCy (72.3 ± 7.5% and 67.4 ± 7.6%). We used similar conditioning regimen as Lee et al’s study except the schedule of busulfan. The different results might be attributed to the different disease composition and disease status in these two studies.

## Conclusions

Our randomized prospective trial suggests that BuFlu regimen is a myeloablative conditioning regimen with reduced toxicity. It is well tolerated and possesses similar anti-leukemic activity compared with BuCy in AML-CR1 patients undergoing allo-HSCT.

## Abbreviations

Bu: Busulfan; Flu: Fludarabine; Cy: Cyclophosphamide; allo-HSCT: Allogeneic hematopoietic stem cell transplantation; AML: Acute myeloid leukemia; CR1: First complete remission; RRT: Regimen-related toxicity; GVHD: Graft-versus-host disease; TRM: Transplant related mortality; HVOD: Hepatic veno-occlusive disease; OS: Overall survival; DFS: Disease free survival; MDS: Myelodysplastic syndrome; PBSCs: Peripheral blood stem cells; G-CSF: Granulocyte colony-stimulating factor; CsA: Cyclosporine A; MTX: Methotrexate; ATG: Human anti-thymocyte globulin; MMF: Mycophenolate; CMV: Cytomegalovirus; IFI: Invasive fungal infection; EBV: Epstein barr virus; ANC: Absolute neutrophil count; PTLD: Post-transplant lymphoproliferative disease; RR: Relative risk.

## Competing interests

The authors declare that they have no competing interests.

## Authors’ contributions

HL performed investigations, analyzed data and wrote the paper; XZ, YZ, ZPF and GPY analyzed data; ZYS, JS, YX, DNN, SFT, YHL, FH, HSZ and XTG performed investigations; QFL designed study and wrote the paper. The authors reported no potential conflicts of interest. All authors read and approved the final manuscript.
